# Epigenetic, histological and clinical characterization of preeclampsia in oocyte donation pregnancies: insights into immune dysregulation and microRNA-mediated pathways

**DOI:** 10.3389/fcell.2025.1718305

**Published:** 2026-01-05

**Authors:** Irma Saulle, Maria Di Giminiani, Ozge Yazici, Claudio Fenizia, Manuela Nebuloni, Roberta Rossi, Mara Biasin, Valeria Savasi

**Affiliations:** 1 Department of Biomedical and Clinical Sciences University of Milan, Milan, Italy; 2 Unit of Obstetrics and Gynecology, ASST Fatebenefratelli-Sacco, Department of Biological and Clinical Sciences L. Sacco, University of Milan, Milan, Italy; 3 Department of Pathophysiology and Transplantation, University of Milan, Milan, Italy; 4 Pathology Unit, ASST Fatebenefratelli-Sacco, Milan, Italy; 5 Obstetrics and Gynecology Unit, Buzzi Children’s Hospital, University of Milan, Milan, Italy

**Keywords:** cytokines, inflammation, miRNA, oocyte donation, preeclampsia

## Abstract

**Introduction:**

Preeclampsia (PE) is a hypertensive disorder in pregnancy, influencing global health risks due to its poorly understood aetiology involving immune mismatches. Oocyte Donation increases PE risk due to complete HLA incompatibility, leading to immune activation. MicroRNAs (miRNAs) emerged as crucial regulators in placental development, immune regulation, and endothelial function, acting as post-transcriptional gene regulators. This study aims to explore whether specific miRNAs, previously implicated in PE, can be used to distinguish preeclamptic and non-preeclamptic mothers undergoing oocyte donation pregnancy.

**Methods:**

This prospective study enrolled 20 mothers, divided into four groups: oocyte donation normotensive, oocyte donation preeclamptic, spontaneous normotensive, and spontaneous preeclamptic mothers. Maternal and cord blood samples were collected postpartum, along with placental biopsies. Tissue samples underwent histological examination. Total miRNAs were extracted from plasma, cord blood, and placenta and quantified via digital droplet PCR. The secretome analysis of cytokine/chemokines was performed on the mother’s plasma and cord blood by Luminex ELISA.

**Results:**

In oocyte normotensive the epigenetic (miR-155, miR-17, miR-30) and immune profile (CXCL10, VEGF), displayed only limited variations compared to spontaneous normotensive. Conversely, preeclamptic oocyte recipients exhibited marked molecular dysregulation, characterized by significant upregulation of pro-inflammatory miRNAs (miR-155, miR-17, miR-223) and cytokines (IL-6, IL-1β, TNF-α, IFN-γ) in maternal plasma and placental tissue, indicating heightened immune activation. Notably, miR-30 and let-7c were downregulated. Intriguingly, miRNA expression in umbilical cord plasma was often inversely correlated with maternal and placental profiles, suggesting complex miRNA trafficking and fetal protection mechanisms. Placental histology showed minimal pathological changes in preeclamptic oocyte recipients, contrasting with more severe lesions in preeclamptic spontaneously conceived pregnancies, reflecting differing underlying pathogenic processes.

**Conclusion:**

This study highlights significant alterations in miRNA expression and cytokine profiles associated with PE, particularly in oocyte donation pregnancies. The findings suggested a complex interplay between maternal immune regulation and placental function, with distinct maternal and fetal immune responses. Understanding these molecular and immunological changes may facilitate the development of novel diagnostic biomarkers and targeted therapies to improve maternal and fetal outcomes in PE.

## Graphical Abstract

**Figure:**
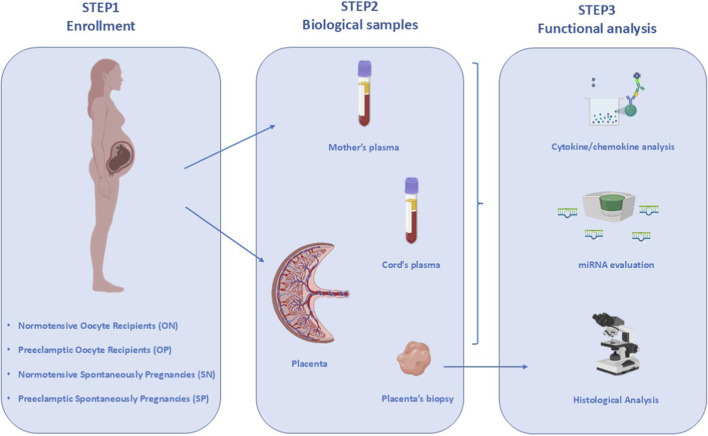

## Introduction

Preeclampsia (PE) is a severe hypertension disorder of pregnancy that significantly influences maternal and fetal health (Chang et al., 2023; Dimitriadis et al., 2023). It is defined by the new development of hypertension (≥140/90 mmHg in a later stage of pregnancy) and proteinuria (≥300 mg of protein) or other signs of organ system complications that appear after 20 weeks of pregnancy (Roberts et al., 2021; Magee et al., 2022; Dimitriadis et al., 2023). On a global scale, PE has an occurrence rate of 2%–8%, which contributes towards it being one of the primary factors for the high mortality and morbidity in mothers and infants (Poon et al., 2019; Magee et al., 2022). Despite extensive research, the exact aetiology of PE is still poorly understood, although it is increasingly recognized that a mismatch of the immune system at the interface between mother and child plays a crucial role in its development (Staff, 2019; Zhang et al., 2020; Yang et al., 2021; Kornacki et al., 2024).

The incidence of PE is markedly increased in pregnancies following the use of oocyte donation (OD) (Blázquez et al., 2016; Berntsen et al., 2021; Keukens et al., 2022). Among the various assisted reproductive technologies (ART), OD pregnancies are particularly challenging from an immunological perspective because of high level of HLA mismatch between fetus and mother (Tilburg et al., 2009; van Bentem et al., 2024). In natural conception (NC) pregnancies, partial maternal-fetal HLA compatibility helps in immune tolerance; conversely, in OD pregnancies, the fetal genome is completely allogeneic due to the contributions from sperm and oocyte donors (van ‘t Hof et al., 2021). This leads to increased maternal immune activation and thus increases risk of PE, implicating inadequate immune matching and impaired immune adaptation as a key contributor to disease development (Martínez-Varea et al., 2014; van Bentem et al., 2020; Zhuang et al., 2021). The role of microRNAs in placental development (Hayder et al., 2018; Xu et al., 2021; Jin et al., 2022), immune modulation (Jia and Wei, 2020; Saulle et al., 2021; Saulle et al., 2023; Gaál, 2024), and endothelial function (Fernández-Hernando and Suárez, 2018; Hromadnikova et al., 2020; Yan et al., 2024) has been rising in recent research. MiRNAs are small, non-coding RNAs that act as gene expression regulators at the post-transcriptional level by targeting mRNAs for degradation or translation repression (Bartel, 2004; de Mello et al., 2024; Hynes and Kakumani, 2024). Several placenta miRNAs have been shown to regulate maternal immune tolerance (Bidarimath et al., 2014; Kamity et al., 2019; Guo et al., 2025). These miRNAs regulate key immune cell populations by modulating the function of natural killer cells, decidual natural killer (NK) cells (Leong et al., 2014; Li et al., 2021), and by activating dendritic cells (Della Bella et al., 2011; Scalavino et al., 2020). They also influence T-cell responses (Chen et al., 2023) and the polarization of decidual macrophages (Zhang et al., 2022). Through these coordinated actions, miRNAs contribute to establishing the immune environment required for a successful pregnancy (Giannubilo et al., 2024). Disruptions in the regulatory networks involving miRNAs may result in maternal immune activation, systemic inflammation and impaired placentation that may lead to PE (Chen et al., 2021). Furthermore, it has been suggested that circulating miRNAs may be used as diagnostic biomarkers of normal and/or pathological pregnancies based on their stability in maternal blood and their ability to reflect placental dysfunction (Kim et al., 2020; Gerede et al., 2025; Munaut et al., 2016).

In this prospective study, our aim is to explore whether miRNAs previously implicated in PE are expressed in both preeclamptic and non-preeclamptic mothers undergoing OD. This profiling may provide valuable insights into understanding the role of miRNAs in the pathogenesis of PE, immune regulation within OD pregnancies, the underlying disease mechanisms and facilitate the development of targeted therapeutic strategies to modulate immune responses and improve maternal-fetal outcomes.

## Methods

### Study population

This prospective, single-centre study included 20 pregnant patients, categorized into four groups based on the mode of conception and the presence or absence of preeclampsia as follows:

- Normotensive Oocyte Recipients (ON)
- Preeclamptic Oocyte Recipients (OP)
- Normotensive Spontaneously Conceived Pregnancies (SN)
- Preeclamptic Spontaneously Conceived Pregnancies (SP)

All participants were admitted to the delivery unit of Vittore Buzzi Maternity Hospital in Lombardia, Italy, between September 2023 and June 2024. The hospital is a Level III Obstetric Center, the third largest maternity unit in Lombardy, Italy’s most populous geographical area. Demographic and anthropometric data, along with medical and obstetric comorbidities, were collected at the time of enrolment using a customized data collection form. All pregnancies were monitored regularly until delivery. Eligible participants were women over 18 years of age, of Caucasian ethnicity, with a singleton pregnancy, receiving prenatal care at Buzzi Hospital in Milan, and who had provided written informed consent.

The diagnosis criteria of PE were complied with the ACOG Practice Bulletin in 2019 DOI: 10.1097/AOG.0000000000003018. PE patients who had gynecological diseases or tumors, multiple pregnancies, chronic hypertension and heart or liver or kidney disease were excluded. All PE patients had mild PE: maternal systolic blood pressure exceeding 140 mmHg and/or diastolic blood pressure exceeding 90 mmHg, with significant proteinuria (≥300 mg of protein in a 24-h urine specimen or ≥ 1+ by dipstick). Healthy pregnant women who had no history of PE or any other complications, such as preterm delivery, stillbirth, fetal malformations, maternal hypertension, genetic abnormalities, autoimmune diseases, or a history of kidney, systemic lupus erythematosus, and maternal infection were included. Exclusion criteria comprised individuals under 18 years of age or non-Caucasian ethnicity, multiple or unattended pregnancies, pre-existing maternal medical conditions and active smoking. Several patients received prophylactic low-dose aspirin (LDA) due to an increased risk of preeclampsia identified through clinical history or first-trimester screening. Specifically, 8 patients in the OD group and 3 in the spontaneous-conception group were treated with LDA.

The study protocol was approved by the local Medical Ethics and Institutional Review Board (Milan, Area 1, protocol no. 32923/2019). Written informed consent was obtained from all participants in accordance with the principles of the Declaration of Helsinki.

### Plasma and placenta biopsy collection

Blood samples from pregnant women scheduled for elective caesarean section were collected after hospital admission and prior to the procedure. For healthy controls, blood samples were collected a few days (3–5 days) before the expected due date. After delivery, 3 mL of cord umbilical blood in EDTA, and full-thickness placental biopsies from the central region of the placenta were obtained by a gynaecologist specifically trained in the appropriate sampling technique and biopsy preservation.

Prior to the biopsy being taken, the placenta was delicately washed by a sterile physiological solution to remove any maternal blood. In order to preserve RNA stability, biological samples from obstetrics and gynaecology units were immediately conveyed to the laboratory of immunology, University of Milan, to be processed or stored. Plasma was obtained by centrifugation of whole blood and cord blood. For histological analysis, all placentas were stored and examined at the Pathology Unit of Luigi Sacco Hospital, ASST Fatebenefratelli Sacco, Milan.

### Macroscopic and microscopic examination of histologically stained placental tissues

Among 20 cases, 13 placentas, ONs (3), OPs (4), SNs (2), SPs (4) were sent to the Department of Pathology of Sacco Hospital and formalin fixed. Sampling was made following the Amsterdam Consensus Statement (Khong et al., 2016). Briefly, each placenta was weighed and measured. Every parenchymal lesion was described and measured. For (Khong et al., 2016) histological purposes, a minimum of four blocks from every placenta were taken: one membrane rolls and two cross sections of umbilical cord (in the same block), and a minimum of three blocks of full thickness parenchyma were taken, one from the cord insertion on the placental disk and the others from normal parenchyma. Each different lesion, if present, was measured and sampled.

Samples were paraffin embedded and for each block a haematoxylin and eosin stain was performed (https://doi.org/10.1016/j.biocel.2025.106880). Each case was reviewed by two experienced pathologists (MN and RSR). The diagnoses were made following Amsterdam Consensus Criteria (Khong et al., 2016). If necessary, haematoxylin and eosin (H/E) was carried out in order to define the nature of the inflammatory infiltrate.

### miRNA extraction from plasma and placenta biopsy

Total miRNAs from mother’s plasma, cordon plasma (100 μL) and placenta were semi-automatically extracted using miRNA serum/plasma kit and microRNA tissue extraction respectively (Promega, Fitchburg, WI, United States) and extracted through the Maxwell® RSC Instrument (Promega, Fitchburg, WI, United States) according to manufacturer’s protocol (Saulle et al., 2023); for all samples an equal concentration of extracted miRNAs was retro-transcribed in cDNA (miRCURY LNA RT kit, Qiagen) (Saulle et al., 2019). To avoid variations due to sample differences and handling, all the variables involved in the procedure were kept consistent throughout the study.

### Quantification of mother’s and cordon’s plasma and placenta miRNAs

The analysis of 10 plasma and placenta miRNAs was performed on miRNAs extracted from serum of 5 OPs, 5 NOs, 5 SNs and 5 SPs. Selected miRNAs were analysed by droplet digital PCR (ddPCR QX22, Bio-Rad) in all the individuals enrolled in the study. Three μl of diluted cDNA (1:50) were mixed with ddPCR EvaGreen Supermix (Bio-Rad), LNA™ specific primers (Qiagen), and subsequently emulsified with droplet generator oil (Bio-Rad) by QX200 droplet generator (Bio-Rad). Droplets were transferred to a 96-well reaction plate and heat-sealed with a pierceable sealing foil sheet by a PCR plate sealer (PX1, Bio-Rad). PCR amplification was performed in sealed 96-well plate using a T00 thermal cycler (Bio-Rad) as follows: 10′ at 95 °C, 40 cycles at 94 °C for 30″ and 58 °C for 60″, followed by 10′ at 98 °C and a hold at 4 °C. The plate was then transferred to a QX200 droplet reader (Bio-Rad) (Cappelletti et al., 2025). Each well was queried for fluorescence to determine the quantity of positive events (droplets), and the results were displayed as dot plots. The miRNA concentration was expressed as copies/µL.

### Cytokine and chemokine multiplex analysis

A 27-cytokine multiplex assay was performed in maternal and cord blood plasma of all participants included in the study using magnetic bead immunoassays according to the manufacturer’s protocol (BioRad, Hercules) (Saulle et al., 2025). Arbitrary concentrations of 4,000 pg/mL and 0.1 pg/mL were assigned to values respectively over or below the detection limit.

### Statistical analyses

The Student’s t-test was done when appropriate for statistical analysis to compare continuous and categorical variables. One-way ANOVA were applied for parametric and non-parametric comparisons. A *p*-value < 0.05 was chosen as the cut-off for significance. Results were expressed as mean ± SEM. All statistical analyses and graphs were performed with GraphPad Prism 10.4.2 (GraphPad Software, San Diego, CA, United States).

## Results

### Population

The main characteristics and pregnancy outcomes of the study population were summarized in Table 1. The mean maternal age differed significantly between the spontaneous and oocyte donation groups and was 36 years for patients with SPs and 46 years for those with OD. The pre-pregnancy BMI was 25 in this population, slightly higher in OD groups than SP groups, with no statistically significant differences. All participants across the subgroups were Caucasian and exhibited similar and comparable baseline characteristics, were in good general health, and without any pre-pregnancy clinically significant comorbidities.

**TABLE 1:** Baseline characteristics and pregnancy outcomes of the study population.

Cohort characteristics	Spontaneous conception -SN-(*n* = 5)	Oocyte donation-ON-(*n* = 5)	Spontaneous conception with preeclampsia-SP-(*n* = 5)	Oocyte donation with preeclampsia-OP-(*n* = 5)
Maternal age, years, *mean (DS)*	34.8 (4.9)	46.8 (2.9)	36.3 (6.3)	45.2 (1.3)*
Pre-pregnancy BMI, kg/m^2^, *mean (DS)*	23.5 (5.2)	28.5 (6.9)	25.5 (4.6)	25.0 (5.6)
Gestational age at delivery, days, *mean (DS)*	281 (11.6)	268 (1.4)	266 (13.3)	268 (5.8)*
Delivery mode				
Spontaneous vaginal delivery, *n (%)*	4 (80)	0	0	1 (20)
Caesarean section (CS), *n (%)*	1 (20)	5 (100)	5 (100)	4 (80)
Elective CS, *n (%)*	0	5 (100)	3 (60)	4 (80)
Urgent CS, *n (%)*	1 (20)	0	2 (40)	0
Other pregnancy complications				
Preterm birth, *n (%)*	0	0	1 (20)	0
Gestational diabetes, *n (%)*	0	1 (20)	2 (40)	0
Fetal growth restriction, *n (%)*	0	0	2 (40)	1 (20)
Maternal blood loss, mL, *mean (DS)*	700 (353)	300 (81)	450 (238)	430 (239)
Placental weight, g, *mean (DS)*	576 (85)	434 (120)	329 (120)	359 (63)*
Neonatal weight, g, *mean (DS)*	3,350 (359)	3,210 (417)	2,800 (757)	2,863 (354)
Umbilical artery pH, *mean (DS)*	7.29 (0.14)	7.33 (0.07)	7.30 (0.08)	7.28 (0.11)
APGAR score 5’ <7, *n (%)*	0	0	0	0

*Gestational age at delivery and placental weight differed significantly between spontaneous pregnancies without preeclampsia and other groups (*p ≤* 0.05). No other differences were found (one-way ANOVA), except for maternal age between oocyte donation and spontaneous pregnancies (t-test).

Most of the patients delivered between 38 and 39 weeks of gestation, with only one preterm delivery at 35 weeks due to severe preeclampsia and fetal growth restriction in SPs with preeclampsia cohort. Other obstetric complications found were fetal growth restriction and gestational diabetes. Vaginal delivery was common for the SP patients, whereas in the other groups, most patients underwent elective caesarean sections due to either pregnancy-related complications or maternal request. Maternal blood loss was higher in the SP group, with only one severe maternal haemorrhage (>1,000 mL). There were no significant differences in neonatal outcomes. The mean birth weight was 3,061 g, with a small and non-significant difference between PE and non-PE cohorts. APGAR scores and umbilical cord pH were within normal ranges for all neonates.

### Placental histological findings in women diagnosed with and without preeclampsia

**FIGURE 1:**
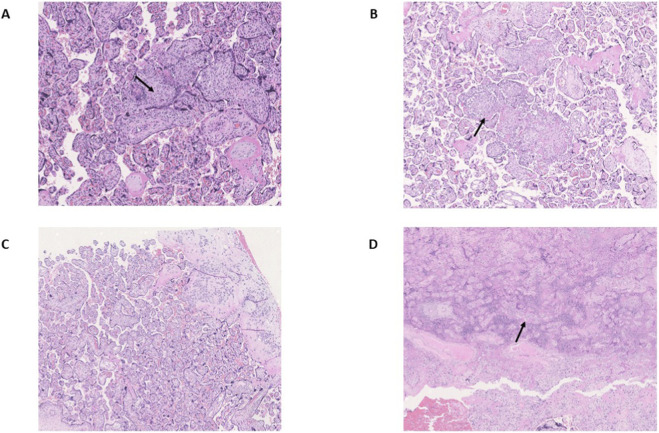
Photomicrographs of placental changes observed among pregnant women from the study. **(A)** OPs placentas show high-grade lymphohistiocytic villitis (VUE) with collapsed villi and destroyed syncytiotrophoblast affected in over 10 contiguous terminal villi (EE 20×). **(B)** ONs placentas display low-grade lymphohistiocytic VUE with fewer than 10 affected villi (EE 20×). **(C)** SNs placentas exhibit normal villous architecture with no ischemic or inflammatory changes. **(D)** SPs placentas demonstrate recent ischemic damage, characterized by collapsed necrotic villi typical of placental infarction (EE 20×).

Among 20 placentas, 13 were formalin fixed and paraffin embedded and were subdivided into 4 groups. In the OP group, there were two cases with an anomalous cord insertion. The placental weight was under the 10th percentile in one case. There were two cases (50%) with pathological histology: one maternal vascular malperfusion and one with High Grade lymphocytolytic villitis (Figure 1A).

In the ON group (3 placentas) showed two cases with a pseudo normal cord insertion and one case was not evaluable. No macroscopic lesions were observed. Histologically only one case (33%) was pathological, showing a low grade lymphocytolytic villitis (Figure 1B).

In the SP group (4 placentas) showed one case with an anomalous cord insertion, two cases with a placental weight under the third percentile and with macroscopic lesions. Three cases (75%) showed a pathological histology (maternal vascular malperfusion).

In the SN group (2 placentas) no anomalous cord insertion was observed, the weight of one placenta was under 10th percentile and no pathological histology was observed (Figure 1C).

Overall unique pathological features were observed and reported by histological observation mainly from the placentas of SPs’ pregnancies (Figure 1D).

### MiRNA expression in maternal plasma

**FIGURE 2:**
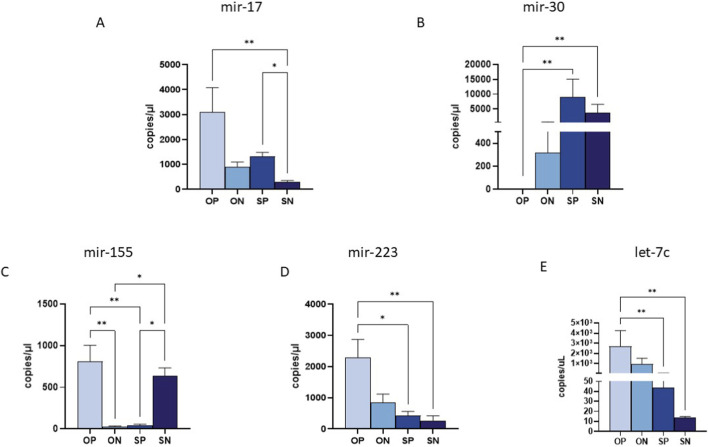
miRNA quantification in maternal plasma samples by ddPCR. Quantification of the expression (copies/uL) of miR-17 **(A)**, miR-30 **(B)** and miR-155 **(C)**, miR-223 **(D)** and miRlet-7c **(E)** in maternal plasma samples. Values were mean ± SEM. Significance was indicated as follows: *p < 0.05, and **p < 0.01.

Of the 10 analysed miRNAs (miR-17, miR-30, miR-140, miR-146, miR-150, miR-155, miR-181a, miR-210, miR-223 and let-7c), only five exhibited significantly different expression levels in maternal plasma collected from ONs, OPs, SNs and SPs (Figures 2A–E). Specifically, miR-17 showed an upregulation in OPs compared to all other groups, with the highest significance observed in comparison to SNs (*p* ≤ 0.01), and in SPs compared to SNs (*p* ≤ 0.05) (Figure 2A). Conversely, miR-30 displayed a pronounced downregulation in OPs across all comparisons, reaching statistical significance in the OPs compared to the SPs and SNs (OPs vs. SPs *p* ≤ 0.01; OPs vs. SNs *p* ≤ 0.01) (Figure 2B).

Regarding the miRNAs involved in immune system regulation, we observed a significant upregulation of anti-inflammatory miRNAs such as miR-155 and miR-223 in the plasma of OPs compared to the other analysed groups (Figures 2C,D). Specifically, miR-155 was highly expressed in OPs, with significant differences observed when compared to both SPs and ONs (OPs vs. ONs: *p* ≤ 0.01) (Figure 2C). Additionally, SNs exhibited high miR-155 expression compared to both SPs and ONs (*p* ≤ 0.05 for all comparisons) (Figure 2C). Similarly, miR-223 was significantly upregulated in OPs compared to SPs (*p* ≤ 0.05) and SNs (*p* ≤ 0.01) (Figure 2D). Finally, miR-let-7c showed reduced expression in SNs compared to both SPs and OPs (*p* ≤ 0.01) (Figure 2E).

No statistically significant differences were observed for the other five miRNAs previously associated with preeclampsia, namely miR-140, miR-146, miR-150, miR-181a, and miR-210.

### miRNAs expression in the placenta

**FIGURE 3:**
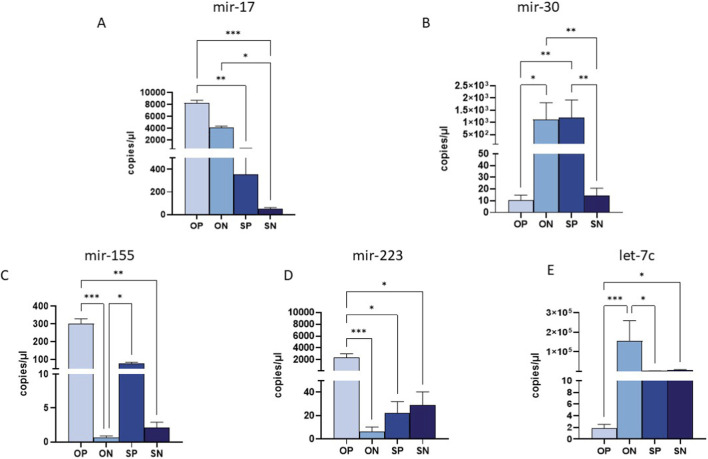
miRNA quantification in placental biopsy by ddPCR. Quantification of the expression (copies/uL) of miR-17 **(A)**, miR-30 **(B)** and miR-155 **(C)**, miR-223 **(D)** and miR-let-7c **(E)** in maternal plasma samples. Values were mean ± SEM. Significance was indicated as follows: *p < 0.05, **p < 0.01 and ***p < 0.001.

The 10 selected miRNAs were expressed in placental tissue from all enrolled groups. However, analogously to what was observed in maternal plasma, significant differences were observed in the expression of only 5 miRNAs, particularly by comparing OPs to the other three groups. In detail, the expression of miR-17 was significantly upregulated in OPs compared to SPs (*p* ≤ 0.01) and SN (*p* ≤ 0.001) (Figure 3A); in addition, there was also a significant upregulation in ONs vs. SNs (*p* ≤ 0.05) (Figure 3A). Conversely, miR-30 was markedly downregulated in OPs and SNs compared to both ONs and SPs (OPs vs. ONs *p* ≤ 0.05; OPs vs. SPs: *p* ≤ 0.01; SNs vs. SPs: *p* ≤ 0.01) (Figure 3B). Moreover, we also observed a drastic upregulation of miR-30 in ON vs. SN: *p* ≤ 0.01 (Figure 3B). Mir-155 displayed significantly higher expression in OPs compared to ONs and SNs (OP vs. ON: *p* ≤ 0.001; OP vs. SN: *p* ≤ 0.01) (Figure 3C). High levels of miR-155 were also observed in SPs compared to ONs (*p* ≤ 0.05 for all comparisons) (Figure 3C). Furthermore, miR-223 was significantly upregulated in OPs compared to ONs, SPs and SNs (OP vs. ON: *p* ≤ 0.001; OPs vs. SPs and SNs: *p* ≤ 0.01 for both comparisons) (Figure 3D). A substantial reduction in let-7c expression was observed in OPs compared to ONs and SNs (OPs vs. ONs *p* ≤ 0.001; OPs vs. SNs *p* ≤ 0.05), as well (Figure 3E).

### MiRNA expression in umbilical cord plasma

**FIGURE 4:**
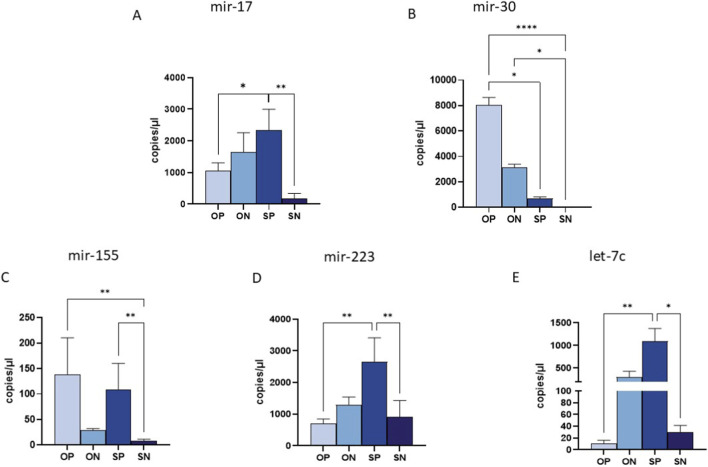
miRNA quantification in Umbilical cord plasma by ddPCR. Quantification of the expression (copies/uL) of miR-17 **(A)**, miR-30 **(B)** and miR-155 **(C)**, miR-223 **(D)** and miR-let-7c **(E)** in maternal plasma samples. Values were mean ± SEM. Significance was indicated as follows: *p < 0.05, **p < 0.01 and ****p < 0.001.

MiRNA expression in umbilical cord plasma revealed significant differences compared to both maternal plasma and placental tissue, underscoring unique maternal-fetal miRNA dynamics. In fact, miR-17, which was significantly upregulated in the maternal plasma and placenta of OPs, showed marked downregulation in the umbilical cord plasma of newborns from OPs compared to SPs (*p* ≤ 0.05) (Figure 4A) and in SNs compared to SPs (*p* ≤ 0.01) (Figure 4A). Similarly, miR-30, which exhibited downregulation in the maternal plasma of OPs, was found to be significantly upregulated in the cord blood plasma of newborns from OPs when compared to both SPs and SNs (OPs vs. SPs: *p* ≤ 0.05; OPs vs. SNs: *p* ≤ 0.001) (Figure 4B). In addition, miR-30 showed a higher expression in ONs compared to SNs (*p* ≤ 0.05) (Figure 4B). Analogous trends were observed for miR-155, miR-223 and let-7c, with a marked reduction in OPs’ cord plasma compared to SPs (Figures 4C–E). However, statistical significance was reached only for miR-223 and let-7c (*p* ≤ 0.01 for both) (Figures 4D,E). In addition, miR-155, miR-223 and let-7c were highly expressed in cord blood of newborns from SPs compared to SNs (miR-155: *p* ≤ 0.01; miR-223: *p* ≤ 0.01; let-7c *p* ≤ 0.05) (Figures 4C–E). In addition, miR-155 showed an increased expression in OPs compared to SNs (*p* ≤ 0.01) (Figure 4E). This inverse expression pattern suggests a potential compensatory mechanism or specific miRNA trafficking between the maternal and fetal compartments.

### Cytokine expression in maternal and cord blood plasma

**FIGURE 5:**
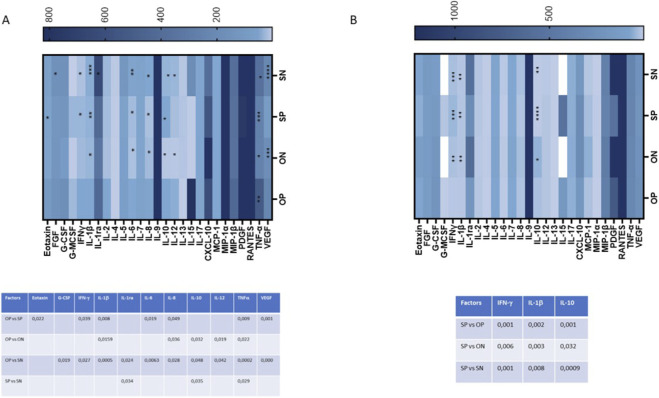
Plasma secretion of cytokines/chemokines that were part of the inflammatory response. The production of 27 cytokines/chemokines regulating immune response was assessed by Luminex assay in mother’s plasma **(A)** and umbilical cord plasma **(B)**. Cytokine/chemokine productions (mean values) are shown as a colour scale from white to blue (heatmap). Only statistically significant p-values between at least 2 groups were reported in lower panels of (**A**, mother’s plasma) and (**B**, umbilical cord plasma).

The production of 27 cytokines and chemokines involved in immune activation was assessed in both maternal and cord blood plasma from all patients using a Luminex assay. Overall, cytokine profiles differed markedly between the two anatomical compartments. Maternal plasma from OPs pregnancies exhibited a broadly pro-inflammatory signature compared to all other groups (Figure 5A).

In particular, several cytokines implicated in preeclampsia pathogenesis (Raghupathy, 2013) were significantly upregulated in OPs’ maternal plasma (Figure 5A). Notably, increased levels were observed in OPs vs. SPs for Eotaxin (*p* ≤ 0.05), IFN-γ (*p* ≤ 0.05), IL-1β (*p* ≤ 0.01), IL-6 (*p* ≤ 0.05), IL-8 (*p* ≤ 0.05), TNF-α (*p* ≤ 0.01), and VEGF (*p* ≤ 0.01) (Figure 5A). OPs vs. ONs comparisons revealed elevated levels of IL-1β (*p* ≤ 0.05), IL-8 (*p* ≤ 0.05), IL-10 (*p* ≤ 0.05), IL-12 (*p* ≤ 0.05), and TNF-α (*p* ≤ 0.05). Similarly, OPs vs. SNs showed significant increases in G-CSF (*p* ≤ 0.05), IFN-γ (*p* ≤ 0.05), IL-1β (*p* ≤ 0.001), IL-1ra (*p* ≤ 0.05), IL-6 (*p* ≤ 0.01), IL-8 (*p* ≤ 0.05), IL-10 (*p* ≤ 0.05), IL-12 (*p* ≤ 0.05), TNF-α (*p* ≤ 0.001), and VEGF (*p* ≤ 0.001) (Figure 5A).

By contrast, only a limited number of cytokines were upregulated in the SPs compared to others. Specifically, SPs vs. SNs showed increased levels of IL-1ra (*p* ≤ 0.05), IL-10 (*p* ≤ 0.05), and TNF-α (*p* ≤ 0.05) (Figure 5A).

This pro-inflammatory pattern was not reflected in the cord blood plasma (Figure 5B). Only three cytokines—IFN-γ, IL-1β, and IL-10—were significantly upregulated, and exclusively in the SPs compared to the SNs (IFN-γ: *p* ≤ 0.001; IL-1β: *p* ≤ 0.01; IL-10: *p* ≤ 0.001), to the ONs (IFN-γ: *p* ≤ 0.01; IL-1β: *p* ≤ 0.001) and compared to the OPs (IFN-γ: *p* ≤ 0.001; IL-1β: *p* ≤ 0.001; IL-10: *p* ≤ 0.001) (Figure 5B).

## Discussion

Despite the complete genetic heterogeneity between the mother and the fetus in normotensive oocyte recipients, our findings indicate that these pregnancies do not exhibit a distinctive placental morphology, epigenetic and immunological profile. Our analysis reveals that, in healthy pregnancies, miRNA expression profiles are highly comparable between oocyte recipients and women with spontaneously conceived conception. Indeed, the miRNA analysed in all anatomical districts do not show significant differences between these two groups, suggesting that, unexpectedly, in pregnancies with a normal course, oocyte donation does not seem to influence the epigenetic profile. The only exceptions are represented by a significant downregulation of miR-155 in maternal plasma and a significant overexpression of miR-17 in the placenta and miR-30 in cord-blood, compared to the spontaneous conception group. Similarly, cytokine profiling revealed minimal variations, with only slight increases of CXCL10 and VEGF in the maternal plasma of oocyte donation pregnancies. These findings suggest that oocyte donation does not substantially alter the normal immune mediator balance during healthy pregnancy. This is further supported by placental histological analysis, which showed no significant differences between healthy oocyte recipients and women with spontaneously conceived, uncomplicated pregnancies. Placentas from both groups appeared structurally normal, with no signs of vascular malperfusion, villitis, or other pathological changes, indicating that in the absence of complications, oocyte donation does not adversely affect placental development or the molecular and immunological profile of pregnancy.

However, the landscape shifts markedly in women developing preeclampsia. Indeed, in our results, preeclamptic oocyte recipients exhibit a highly disrupted miRNA and cytokine profile both in the placenta and maternal plasma, indicative of an intricate immunological dysregulation potentially driven by specific miRNAs.

In particular, miR-17 and miR-155, which are overexpressed in preeclamptic spontaneously conceived pregnancies (Chen et al., 2021; Ntsethe and Mackraj, 2022; Wang et al., 2023; Ng et al., 2024), were significantly upregulated in preeclamptic oocyte donation recipients, both in plasma and placental tissue. In detail, miR-155 induces endothelial activation and influences the production of inflammatory cytokines and the regulation of T cells, as reported by O’Connell et al. (2010). This may explain its upregulation alongside key inflammatory cytokines such as IL-6, IL-1β, TNF-α, and IFN-γ in the maternal circulation of preeclamptic patients with oocyte donation, indicating a synergistic loop of immune activation at the maternal-fetal interface. MiR-17 is a promising epigenetic biomarker for the early detection of preeclampsia and other vascular disorders (Wang et al., 2012; Bounds et al., 2017). Indeed, hsa-miR-17-5p is a critical component of the hsa-miR-17-92 cluster whose increased expression has been linked to a reduced aromatase protein expression and an inhibition of trophoblast differentiation, negatively affecting placental vascularization (Wang et al., 2012; Gan et al., 2017).

A notable observation is that miR-223 is also upregulated both in plasma and placental tissue in oocyte donation pregnancies. MiR-223 is a microRNA which intervenes in different processes including infections (Yahyaei et al., 2016; Saulle et al., 2021; Saulle et al., 2023), inflammation (Haneklaus et al., 2013; Saulle et al., 2021; Das and Rao, 2022) and preeclampsia, via NLRP3 targeting (Haneklaus et al., 2013; Liu et al., 2024). Its increased expression in these patients may, thus, represent a compensatory mechanism to counteract excessive inflammation.

In contrast, miR-30 and let-7c are significantly downregulated in both maternal plasma and placenta from oocyte recipients. This observation aligns with evidence highlighting miR-30’s role in maintaining endothelial homeostasis and regulating immune cell activation (Fernández-Hernando and Suárez, 2018; Yan et al., 2024). Specifically, miR-30 is crucial for reversing the epithelial-mesenchymal transition and reducing cell invasion, plasticity, and VEGF production in epithelial ovarian carcinoma cells (Sestito et al., 2016). Since preeclampsia is characterized by hypertension and increased vascular permeability, the ability of miR-30 to modulate VEGF levels further underscores its potential relevance in the pathophysiology of this condition (Shah and Khalil, 2015).

The downregulation of let-7c is particularly relevant because the expression of this miRNA has been consistently associated with the regulation of cell proliferation and immune activation (Boyerinas et al., 2010; Erice et al., 2024; 2024). Specifically, let-7c targets RAS (Johnson et al., 2005), whose suppression prevents uncontrolled cell growth (Ma et al., 2021), and STAT3 (Signal Transducer and Activator of Transcription 3) (Guo et al., 2013; Patel et al., 2014), thus limiting immune cell activation and proliferation (Hillmer et al., 2016). A reduction in let-7c levels can disrupt these regulatory pathways, resulting in uncontrolled cell proliferation and heightened immune activation. These alterations may underlie the inflammatory and immune dysregulation observed in oocyte donation pregnancies with preeclampsia.

In contrast, histological analyses show that placentas from preeclamptic women with spontaneous pregnancies exhibit significantly more severe pathology than those from preeclamptic oocyte recipients. While the latter typically present with only mild inflammatory infiltrates and villitis, the former often display extensive infarctions and marked tissue damage. Interestingly, this contrasts with molecular findings, as both plasma and placental profiles in oocyte donation cases suggest a more severe disease phenotype compared to spontaneous preeclampsia.

This apparent discrepancy may reflect the fact that systemic molecular markers and placental histopathology capture different dimensions of preeclampsia. In oocyte donation pregnancies, molecular profiles reveal a heightened inflammatory response at both maternal and placental levels, yet this is not accompanied by the extensive tissue damage seen in spontaneously conceived preeclamptic cases. The relatively mild histological findings suggest that inflammation in these cases may be more localized or regulated, resulting in immune activation without overt structural injury. In contrast, spontaneously conceived preeclamptic pregnancies often exhibit more advanced and destructive placental pathology, potentially reflecting distinct or prolonged pathogenic mechanisms. These observations underscore the multifactorial nature of preeclampsia and the imperfect correlation between molecular inflammation and histological severity.

From a clinical perspective, this observation may indicate that, in spontaneous pregnancies, preeclampsia primarily arises from abnormalities in placental development and function—such as insufficient trophoblast invasion and compromised uteroplacental perfusion. Conversely, in oocyte recipients, preeclampsia appears to be more strongly associated with maternal factors, including immune maladaptation or altered cardiovascular responses, rather than with intrinsic placental dysfunction.

A particularly novel aspect of this study is the evaluation of miRNA and cytokine expression in umbilical cord plasma, which revealed a distinct and often opposite expression profile compared to maternal and placental compartments. Notably, miR-17, miR-155, and miR-223 were markedly downregulated in the cord blood of preeclamptic oocyte donation cases despite their strong upregulation in maternal plasma and placental tissue. Similarly, miR-30, which was decreased in maternal compartments, was significantly increased in cord plasma. This inverse correlation suggests a complex and possibly compensatory mechanism of miRNA trafficking or selective retention at the maternal-fetal interface, potentially aimed at protecting the fetus from the maternal inflammatory milieu. Such compartmentalization of molecular responses has been proposed in recent studies, highlighting the placenta as a regulatory barrier that modulates immune and molecular signals between the mother and fetus (Paquette et al., 2018). The cytokine profiles further support this anatomical dissociation. While maternal plasma from preeclamptic oocyte donation pregnancies displays a robust inflammatory signature, characterized by elevated levels of IL-1β, TNF-α, and VEGF, the cord plasma remains largely quiescent from an immunological point of view, indicating that the fetal compartment is largely shielded from the heightened maternal inflammation. This discrepancy may reflect an adaptive response to prevent fetal damage and preserve homeostasis during complicated pregnancies.

Consistent with these observations, multiple studies have demonstrated that preeclampsia is accompanied by a systemic pro-inflammatory state marked by elevated IL-6, IL-1β, TNF-α, IFN-γ, and an imbalance in cytokine regulation (Liu et al., 2023; Valencia-Ortega et al., 2019; Ferguson et al., 2016). The cytokine pattern observed in our oocyte-donation cohort—with increased IL-6, IL-1β, TNF-α, CXCL10, and VEGF—parallels these findings and supports the concept that immune activation and endothelial dysfunction are central to the disease process. In particular, the strong upregulation of CXCL10 aligns with data from Kara et al. (2019), while the elevated TNF-α levels correspond to those described by Cackmac et al. (2016) and Pinheiro et al. (2014), confirming its association with disease severity.

The overall profile observed in oocyte-donation pregnancies, comparable to or even exceeding that of spontaneous preeclampsia, suggests that immune maladaptation rather than placental structural damage may play a predominant role in driving disease severity. Moreover, the relative preservation of cytokine homeostasis in the umbilical circulation, consistent with findings by Valencia-Ortega et al. (2019), highlights the placenta’s role as an active regulator of maternal–fetal cytokine exchange, maintaining fetal protection despite intense maternal inflammation.

Despite the comprehensive design of this study, several limitations should be acknowledged. The relatively small sample size, reflecting the difficulty of recruiting well-characterized oocyte donation pregnancies with and without preeclampsia, may limit the generalizability of our findings.

Maternal age also differed significantly between groups, an intrinsic feature of the oocyte donation population that may have contributed to some of the observed molecular variations. Although our results are consistent with the distinct immunogenetic context of oocyte donation, larger and more age-homogeneous cohorts will be needed to better delineate the relative impact of maternal age and conception mode. Another important limitation concerns the mode of delivery, which could not be fully controlled in this cohort. Caesarean section in the Italian healthcare system is performed only when medically indicated, often in the presence of maternal–fetal complications that may independently influence inflammatory and epigenetic profiles. As a result, it is difficult to disentangle the contribution of delivery mode from the underlying pathophysiological processes of preeclampsia.

Furthermore, while aspirin use did not differ significantly between preeclamptic and normotensive patients, its potential modulatory effects on inflammatory and endothelial pathways should be considered when interpreting molecular data. Finally, variations in delivery mode—mainly reflecting routine clinical management of oocyte donation pregnancies—could have introduced additional variability in circulating miRNA profiles. Future studies incorporating pre-delivery sampling or stratified analyses, with larger, multicentre cohorts and standardized clinical parameters will be essential to confirm and expand these observations.

## Conclusion

**FIGURE 6:**
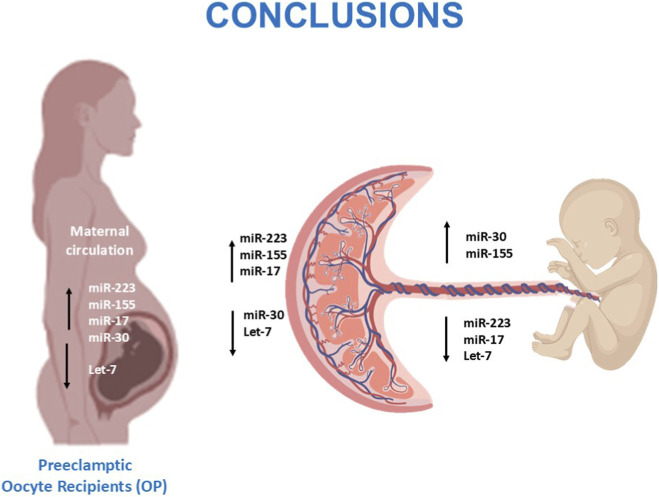
Overview of dysregulated miRNAs in preeclamptic oocyte-recipient pregnancies and their potential effects on the maternal–placental–fetal axis.

This is the first study performing a comprehensive comparison of the epigenetic, immunological and histopathological landscapes in oocyte donation pregnancies and spontaneous conception across healthy and pathological conditions. Our findings indicate that, while oocyte donation pregnancies generally preserve normal placental morphology and immune balance, the onset of preeclampsia is associated with marked molecular dysregulation involving inflammatory miRNAs and cytokine networks. The discrepancy between molecular activation and relatively mild placental lesions suggests distinct mechanisms driving disease expression in oocyte donation compared with spontaneous pregnancies. These results point to a potentially more maternal and immune-mediated origin of preeclampsia in oocyte recipients, contrasting with the predominantly placental pathophysiology in spontaneous cases. Finally, the opposite regulation of miRNAs between maternal and cord compartments highlights an active placental role in modulating fetal exposure to inflammation (Figure 6). Together, these observations underscore the complex and compartment-specific nature of immune regulation in pregnancy.

Given that this is the first study to characterize the epigenetic profile of preeclamptic oocyte donation pregnancies, future research should focus on larger, multicentre cohorts to validate and expand our findings. Longitudinal studies tracking maternal profiles across gestation would provide valuable insights into the temporal evolution of immune and epigenetic alterations in oocyte donation pregnancies. Furthermore, mechanistic studies exploring the functional roles of the identified miRNAs in maternal-fetal immune crosstalk and placental development could reveal potential therapeutic targets. This research may ultimately pave the way for personalized interventions to improve maternal and fetal outcomes in high-risk pregnancies, particularly those involving oocyte donation.

## Data Availability

The raw data will be made available upon request to interested researchers by contacting the corresponding author via email.

## Supplementary material

SUPPLEMENTARY FIGURE S1 Plasma secretion of cytokines/chemokines. Quantification of protein production that reached statistical significance is shown (mean ± SEM). **(A)** Shows significant cytokines/chemokines in maternal plasma, and **(B)** shows those in umbilical cord plasma. Significance is indicated as follows: * p < 0.05, ** p < 0.01, *** p < 0.001.
