# Supplementary figures and images for: Epigenetic, histological and clinical characterization of preeclampsia in oocyte donation pregnancies: insights into immune dysregulation and microRNA-mediated pathways
Source: Front Cell Dev Biol. 2026 Jan 5;13:1718305. doi: 10.3389/fcell.2025.1718305 (PMC12812992; doi:10.3389/fcell.2025.1718305)

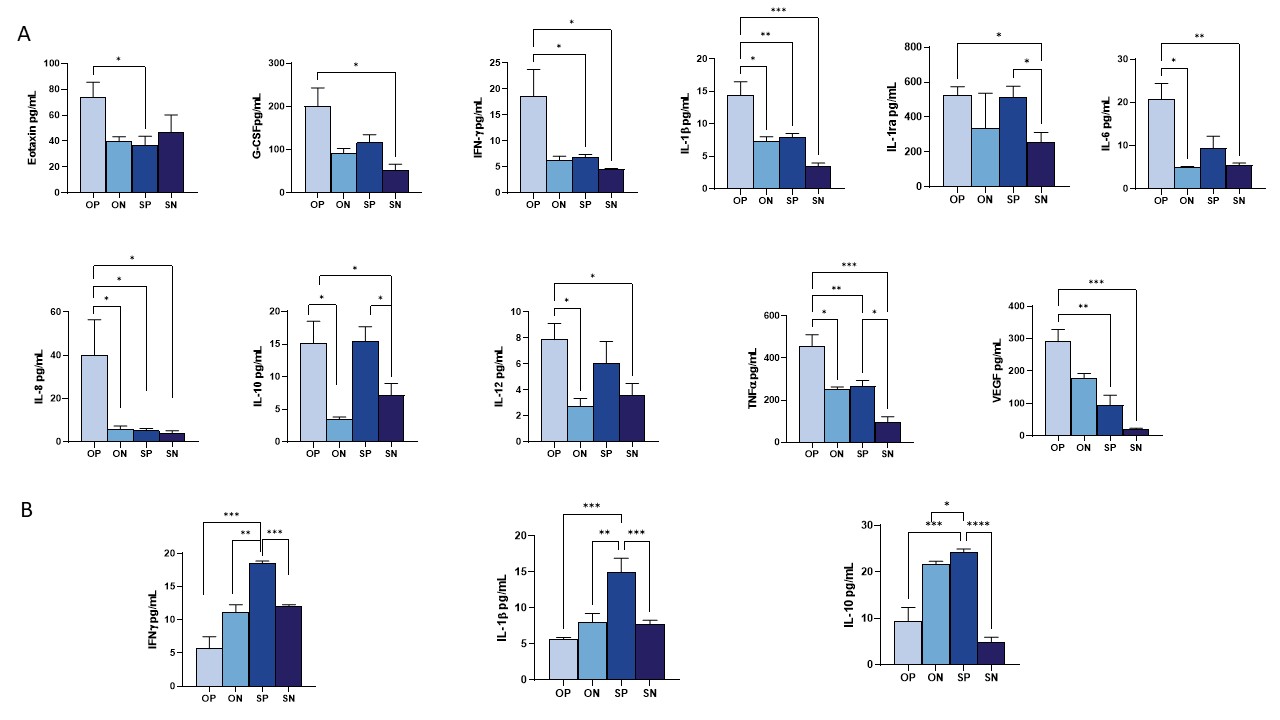

Supplement: Supplementary file 1 [file Image1.jpeg]
